# Assessing the similarity of mental models of operating room team members and implications for patient safety: a prospective, replicated study

**DOI:** 10.1186/s12909-016-0752-8

**Published:** 2016-08-31

**Authors:** Ivana Nakarada-Kordic, Jennifer M. Weller, Craig S. Webster, David Cumin, Christopher Frampton, Matt Boyd, Alan F. Merry

**Affiliations:** 1 Centre for Medical and Health Sciences Education, School of Medicine, University of Auckland, Private Bag 92019, Auckland, 1142 New Zealand; 2 Auckland City Hospital, Auckland, New Zealand; 3 Department of Anaesthesiology, School of Medicine, University of Auckland, Auckland, New Zealand; 4 Department of Medicine, Christchurch School of Medicine and Health Sciences, University of Otago, Christchurch, New Zealand; 5 Wellington, New Zealand

**Keywords:** Multidisciplinary teamwork, Shared mental models, Operating room teams, Patient safety

## Abstract

**Background:**

Patient safety depends on effective teamwork. The similarity of team members’ mental models - or their shared understanding–regarding clinical tasks is likely to influence the effectiveness of teamwork. Mental models have not been measured in the complex, high-acuity environment of the operating room (OR), where professionals of different backgrounds must work together to achieve the best surgical outcome for each patient. Therefore, we aimed to explore the similarity of mental models of *task sequence* and of *responsibility for task* within multidisciplinary OR teams.

**Methods:**

We developed a computer-based card sorting tool (Momento) to capture the information on mental models in 20 six-person surgical teams, each comprised of three subteams (anaesthesia, surgery, and nursing) for two simulated laparotomies. Team members sorted 20 cards depicting key tasks according to when in the procedure each task should be performed, and which subteam was primarily responsible for each task. Within each OR team and subteam, we conducted pairwise comparisons of scores to arrive at mean similarity scores for each task.

**Results:**

Mean similarity score for task sequence was 87 % (range 57–97 %). Mean score for responsibility for task was 70 % (range = 38–100 %), but for half of the tasks was only 51 % (range = 38–69 %). Participants believed their own subteam was primarily responsible for approximately half the tasks in each procedure.

**Conclusions:**

We found differences in the mental models of some OR team members about responsibility for and order of certain tasks in an emergency laparotomy. Momento is a tool that could help elucidate and better align the mental models of OR team members about surgical procedures and thereby improve teamwork and outcomes for patients.

**Electronic supplementary material:**

The online version of this article (doi:10.1186/s12909-016-0752-8) contains supplementary material, which is available to authorized users.

## Background

Effective teamwork is essential for patient safety [1–5]. Failures in teamwork and communication are common in the operating room (OR) and often lead directly to compromised patient care and reduced productivity [6–11]. The tasks of the surgical, anaesthetic, and nursing subteams are closely interlinked and inter-dependent [5, 12]. Members of the OR team should have a common understanding of the plan for patient management [13] and of the roles and responsibilities of each individual. However, the composition of OR teams changes frequently, members come from different professional backgrounds, and decisions may be needed under time pressure, sometimes with ambiguous or incomplete clinical information. Differences in understanding of the situation, the plan, and the key roles and responsibilities of individual team members may arise, and may impact on patient outcomes, particularly in crises, when time is severely limited [14, 15].

Humans function on the basis of their personal understanding of their situation at any time, which is likely to be unique and to represent reality to a varying degree. Apparently bizarre accidents can often be explained on the basis of discrepancies between this internal view of the world, and the facts that actually pertained at the time [9, 16]. This internal representation of reality has been named the person’s “mental model” [17]. Within a team, each member will have his or her own mental model of the situation and the plan, and of when and by whom various tasks should be done [18]. The extent to which these models overlap (like the common intersect of several circles on a Venn diagram) has been called the teams’ “shared mental model” [19, 20]. In practice, the degree of overlap or “sharing” may vary between different subsets of the team, and may change, dynamically, over time. There is likely to be a core set of information that must be shared by all key players if teamwork is to be effective on a regular basis [21], and for team members to be able to adapt to unexpected situations and predict each other’s actions and needs [22, 23]. Substantially shared mental models are presumed to be the cognitive basis of the smooth and effortless coordination observed in many expert teams working in high-intensity environments [3] such as the OR.

Research on the extent of sharing, or similarity, of mental models in healthcare teams is scarce. Most prior investigations have been conducted within the military domain [24–27] or in laboratory settings often involving students in pairs exposed to computer simulations [28, 29]. Studies in the OR have tended to focus on individual professional groups or “silos”, such as anaesthetists [30–32].

Therefore, we aimed to explore the degree of similarity between the mental models of members of multidisciplinary OR teams regarding the key tasks in an upcoming surgical procedure. Our specific questions were: 1) to what degree do OR team members share a mental model of *task sequence* (i.e., when the tasks should be done) prior to the procedure; and 2) to what degree do OR team members share a mental model of *responsibility for task* (i.e., who is primarily responsible for each task) prior to the procedure. To this end we began by developing a card sorting tool for quantification of the degree of similarity (or “sharedness”) of mental models concerning 20 key tasks related to two common clinical scenarios.

## Methods

This study was conducted in the context of the Multidisciplinary Operating Room Simulation (MORSim) project, a larger body of research aimed at examining various aspects of teamwork in the OR (Australia and New Zealand Clinical Trials Registry ID 12612001088831).

### Participants

We recruited 20 complete OR teams (comprising 120 healthcare professionals in total) for the MORSim project from general surgical ORs at two large teaching hospitals in the Auckland region. Each team included: a consultant and a junior surgeon (surgical subteam); a consultant anaesthetist or senior anaesthetic fellow and an anaesthetic technician (anaesthetic subteam); and two nurses (nursing subteam). In each case these were team members that worked together from time to time within their ORs.

### Context

We conducted the MORSim project at the University of Auckland’s Simulation Centre for Patient Safety in a simulated operating room. Each team participated in two simulated abdominal cases, presented in random order to control for time of day and order effects. Scenario 1 was a laparotomy for an abdominal stab wound and scenario 2 was a laparotomy for a perforated viscus. We developed a card sorting tool, named Momento, which can be easily customised for other clinical scenarios. Items in Momento for this work were developed from the clinical requirements of the cases presented to participants.

**Fig. 1:**
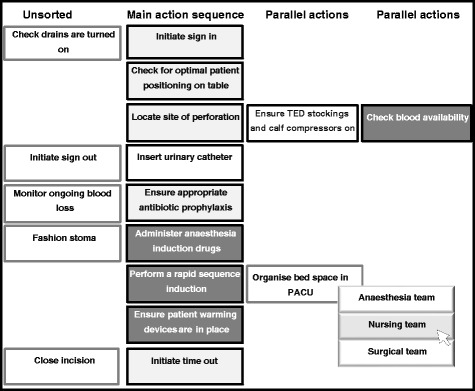
A card sort in progress. A schematic representation of a top left portion of a computer screen showing the card sorting exercise in progress for the laparotomy for a perforated viscus scenario. Participants can sort cards chronologically, down the ‘Main action sequence’ column, and in parallel, by dragging and dropping cards onto the positions in four ‘Parallel Actions’ columns. Whenever a card is dropped onto a position in a column, a menu automatically appears offering a choice of one of the three subteams primarily responsible for the task depicted on that card (as for task ‘Organise bed space in PACU’ above). Teams are colour coded (i.e., dark background with white text = anaesthesia; light grey = surgical; and white = nursing), and the card changes colour accordingly once a team has been assigned to a task

Our approach was informed by several studies [31, 33–35]. In addition, we observed ten relevant clinical cases, and identified a number of tasks for possible inclusion in Momento. We then selected a group of expert OR clinicians, including at least two representatives from each OR professional group (surgery, anaesthesia and nursing). We asked these experts to select tasks relevant to our two study scenarios according to the following criteria: the tasks should be clinically important, and occur routinely in all general surgical laparotomies, and they should have the potential to make a difference to patient care. This produced a list of 20 tasks for each scenario (Table 1), 18 generic and two scenario-specific. These tasks were captured on a set of electronic cards, for presentation in random order on a computer screen. We designed the screen layout with the help of human factors and IT specialists. When using Momento, participants are provided with a summary of an upcoming clinical scenario and are then presented with the relevant set of electronic cards. They are asked to arrange these in the order in which they should be done and to identify the subteam (nursing, surgery or anaesthesia) primarily responsible for ensuring that they are done (as opposed to simply doing the tasks) (Fig. 1). Participants can sort cards in parallel if they think the tasks need to be done at the same time. They can exclude a card, if they believe the associated task is not required for the successful management of the patient in the scenario.

**Table 1:** List of tasks depicted on individual cards in the Momento card sort

Generic tasks	
1. Check blood availability 2. Check for optimal patient positioning on table 3. Initiate sign in 4. Administer anaesthesia induction drugs 5. Perform a rapid sequence induction 6. Ensure appropriate antibiotic prophylaxis 7. Insert urinary catheter 8. Initiate time out 9. Make surgical incision 10. Ensure patient warming devices in place 11. Ensure TED stockings and calf compressors on 12. Monitor ongoing blood loss 13. Organise bed space in PACU (Post-anaesthesia care unit) 14. Close incision 15. Check drains are turned on 16. Confirm estimated blood loss 17. Initiate sign out 18. Provide handover on intraoperative events to PACU staff	
Scenario specific tasks	
Laparotomy for an abdominal stab injury (knife in situ) (scenario 1)	
19. Inform intensive care unit 20. Remove knife from abdomen	
Laparotomy for a perforated viscus (scenario 2)	
19. Locate site of perforation 20. Fashion stoma

We conducted a pilot of the final version of Momento using members of our research team supplemented by several other clinicians, and made adjustments to the software to optimise its ease of use and functionality.

### Conduct of study day

Before starting each case scenario, participants received a demonstration on how Momento computer application works by observing the researcher working through a simple example of a card sort. They were then given a written brief about the upcoming case scenario and the time to read it. Participants were then asked to use Momento to sort the items related to that case. Within each team, participants performed the card sort simultaneously, but independently on individual computers, and without discussion. They were given as much time as they required to complete the card sort. Upon completion of the card sort, participants were shown into the simulated operating room and the scenario commenced.

### Data analysis

For each participant, Momento produces a set of numerical ranks for order of the 20 tasks and a subteam category for responsibility for each task. We conducted all possible pairwise comparisons of individual ranks and categories within the team to calculate the degree of similarity of the mental models.

We calculated similarity scores for the sequence of each task between pairs of subteam members as the absolute difference between the ranks assigned to the task divided by the maximum possible score for any given task (19 is the maximum possible score for any given task, given a list of 20 tasks; a maximum score at this stage of the calculation implies the least possible agreement). We subtracted the result from one. Thus perfect agreement would produce a score of 1–(0/19) = 1, and the worst possible agreement would produce a score of 1–(19/19) = 0. These scores were then expressed as percentages.

We calculated similarity scores for responsibility for each task between pairs of subteam members as ‘1’ if the two participants agreed and ‘0’ if they disagreed and then calculated the mean similarity score for each subteam, expressed as a percentage.

We calculated similarity scores for the multidisciplinary OR team for each task as the mean of all possible pairwise combinations within the OR team. For each task and type of mental model we then calculated the mean similarity score for 20 participating OR teams.

Further details of these calculations can be found in Additional file 1.

## Results

One hundred-and-twenty participants (20 consultant and 20 junior surgeons; 20 consultant anaesthetists or senior anaesthetic fellows and 20 anaesthetic technicians; and 40 nurses) completed the card sort related to scenario 1. One participant, a nurse, was subsequently excluded because she misunderstood the instructions. The card sort for scenario 2 was completed by 119 participants because a junior surgeon had to leave the course early for personal reasons.

Within each OR team, there were more females (62.5 %) than males. Participants’ self-reported clinical experience ranged between 1 (6.7 %) and over 21 years (16 %), and experience in the OR ranged between less than 6 months (2.5 %) and over 21 years (9.2 %). Most participants reported having between 3 and 7 years of clinical (34.2 %) and OR experience (32.5 %).

Participants took an average of 11.34 min (range = 4.46–34.48 min) to complete the card sort for scenario 1, and an average of 13.13 min (range = 3.47–35.10) to complete the card sort for scenario 2.

Tables 2 and 3 show the mean similarity scores for task sequence and responsibility, respectively.

**Table 2:** Mean sequence rank assigned to each task and mean similarity scores on task sequence

Similarity of mental model of task sequence (% agreement)	Scenario 1: Laparotomy for an abdominal stab wound					Scenario 2: Laparotomy for a perforated viscus				
	Mean task rank (in a sequence from 1 to 20)	OR team	Anaesthesia subteam	Surgical subteam	Nursing subteam	Mean task rank (in a sequence from 1 to 20)	OR team	Anaesthesia subteam	Surgical subteam	Nursing subteam
Mean similarity scores for all 20 tasks combined		77	76	80	79		83	83	83	83
1. Check blood availability	2.3	90	88	92	88	2.0	92	93	92	90
2. Check for optimal patient positioning on table	5.6	83	80	89	88	4.9	84	82	85	85
3. Initiate sign in	2.2	90	86	93	93	1.9	91	91	95	91
4. Administer anaesthesia induction drugs	5.4	85	82	85	91	5.1	88	86	88	90
5. Perform a rapid sequence induction	6.0	86	85	88	91	5.5	86	87	86	89
6. Ensure appropriate antibiotic prophylaxis	7.7	86	86	88	85	6.7	84	81	84	86
7. Insert urinary catheter	7.2	86	84	87	85	6.8	85	88	89	82
8. Initiate time out	9.3	85	85	83	89	8.9	86	89	78	88
9. Make surgical incision	12.0	94	93	95	96	11.3	97	96	98	97
10. Ensure patient warming devices in place	6.1	84	83	82	87	5.6	86	86	88	88
11. Ensure TED stockings and calf compressors on	6.3	83	81	88	87	5.4	86	84	89	87
12. Monitor ongoing blood loss	12.3	85	84	83	89	12.5	88	91	84	86
13. Organise bed space in PACU	15.5	80	85	86	79	15.2	81	85	86	76
14. Close incision	15.8	92	92	94	88	15.7	92	93	93	87
15. Check drains are turned on	17.0	89	89	89	92	17.0	90	91	89	92
16. Confirm estimated blood loss	13.1	72	69	74	75	14.5	80	72	82	84
17. Initiate sign out	18.0	87	84	91	89	18.3	91	93	95	88
18. Provide handover on intraoperative events to PACU staff	19.3	92	98	90	86	19.5	95	98	89	98
Inform intensive care unit (scenario1)	8.6	57	66	74	64	n/a	n/a	n/a	n/a	n/a
Remove knife from abdomen (scenario 1)	13.3	92	88	93	92	n/a	n/a	n/a	n/a	n/a
Locate site of perforation (scenario 2)	n/a	n/a	n/a	n/a	n/a	12.3	95	93	97	96
Fashion stoma (scenario 2)	n/a	n/a	n/a	n/a	n/a	14.6	91	93	93	89

**Table 3:** Mean similarity scores for OR team and subteams on responsibility for each task

Similarity of mental model of responsibility for task (% agreement)	Scenario 1: Laparotomy for an abdominal stab wound				Scenario 2: Laparotomy for a perforated viscus			
	OR Team	Anaesthesia subteam	Surgical subteam	Nursing subteam	OR Team	Anaesthesia subteam)	Surgical subteam	Nursing subteam
Mean similarity scores for all 20 tasks combined	69	76	73	72	72	78	73	74
1. Check blood availability	51	55	50	74	55	65	32	65
* 2.* Check for optimal patient positioning on table	39	50	85	37	38	45	79	55
3. Initiate sign in	78	80	85	63	76	80	84	65
4. Administer anaesthesia induction drugs	96	100	95	95	100	100	100	100
5. Perform a rapid sequence induction	100	100	100	100	98	100	95	100
6. Ensure appropriate antibiotic prophylaxis	61	90	55	63	64	90	37	70
7. Insert urinary catheter	55	75	70	68	53	60	84	75
8. Initiate time out	73	70	75	84	73	75	74	75
9. Make surgical incision	100	100	100	100	100	100	100	100
10. Ensure patient warming devices in place	54	80	45	74	55	80	37	80
11. Ensure TED stockings and calf compressors on	83	85	75	84	87	90	68	100
12. Monitor ongoing blood loss	61	90	55	53	58	85	37	45
13. Organise bed space in PACU	69	80	65	74	72	85	79	65
14. Close incision	96	95	100	95	98	100	100	95
15. Check drains are turned on	46	35	55	68	48	45	63	45
16. Confirm estimated blood loss	39	40	50	37	39	55	37	35
17. Initiate sign out	81	85	85	84	77	65	89	75
18. Provide handover on intraoperative events to PACU staff	44	40	45	37	49	45	58	55
Inform intensive care unit (scenario1)	50	70	75	58	n/a	n/a	n/a	n/a
Remove knife from abdomen (scenario 1)	98	100	90	100	n/a	n/a	n/a	n/a
Locate site of perforation (scenario 2)	n/a	n/a	n/a	n/a	98	95	100	100
Fashion stoma (scenario 2)	n/a	n/a	n/a	n/a	97	100	100	90

For the whole team, the overall mean similarity score for both scenarios was 87 % (range 57–97 %, median 86 %) for task sequence. This score exceeded 80 % for all but two tasks in one of the scenarios (Table 2). The overall mean similarity score for both scenarios was 70 % (range 38–100 %, median 70.5 %) for task responsibility and more than half of the items (26 out of 40) scored less than 80 %.

For both scenarios, ‘making a surgical incision’ was the task with the highest mean similarity score both for task sequence and for responsibility (for which the score was 100 %). The lowest mean similarity score for sequence was given for when to inform the intensive care unit during a laparotomy for an abdominal stab wound (scenario 1) (57 %), followed by when to confirm estimated blood loss in both scenarios (72 and 80 %). The lowest score for responsibility was given for checking for optimal patient positioning (38 and 39 %, respectively) closely followed by estimating blood loss (39 % in both scenarios). All participants indicated that the anaesthesia subteam is primarily responsible for performing a rapid sequence induction in scenario 1, and administering anaesthesia induction drugs in scenario 2.

Mean similarity scores on task sequence were largely consistent across the three subteams (see Table 2). Within some subteams mean similarity scores on responsibilities for some of the tasks in the procedure were lower than in other subteams (see Table 3). For example, mean similarity scores were lower among surgical subteams than the anaesthesia and nursing subteams for who should be primarily responsible for ensuring patient warming devices are in place. Similarly, the mean similarity score was higher in anaesthesia subteams than the surgical subteams for who they thought should be primarily responsible for ensuring appropriate antibiotic prophylaxis.

**Fig. 2:**
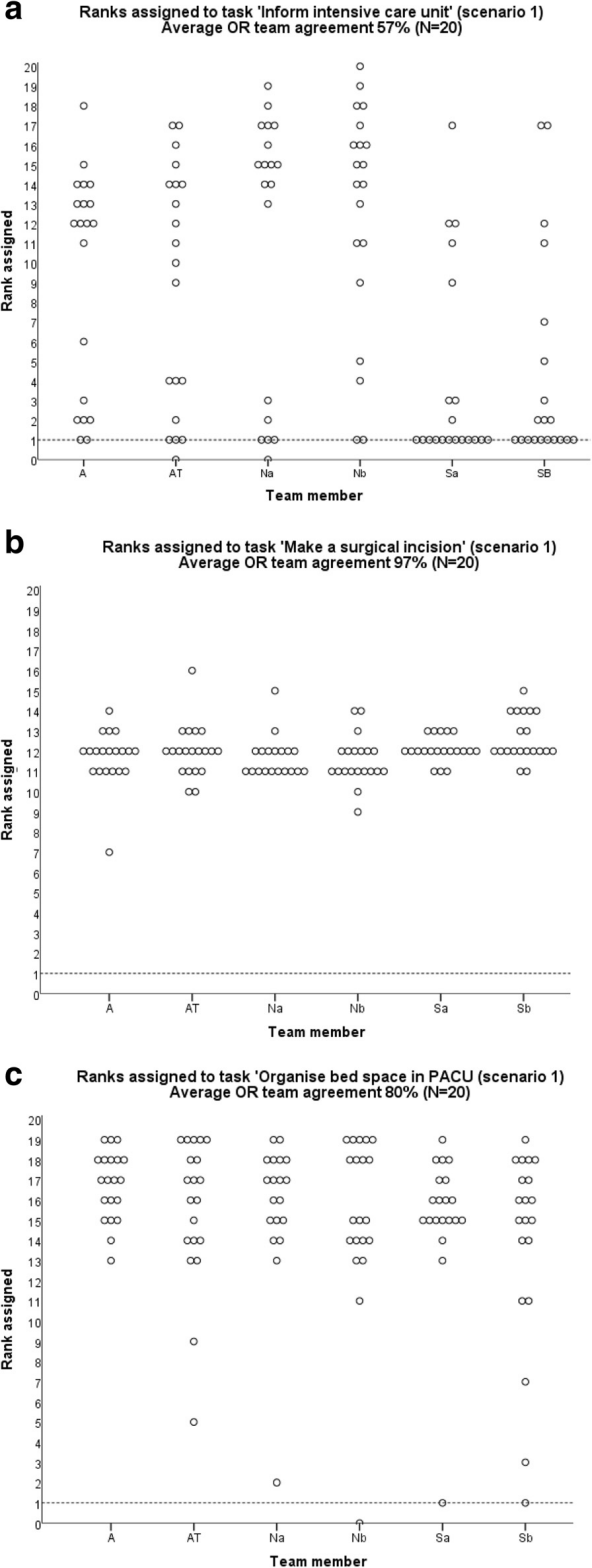
Frequency histograms of ranks assigned by team members to the tasks. The figure depicts frequency histograms with the lowest (**a**), highest (**b**), and mid-range (**c**) OR team agreement (A = anaesthetist; AT = anaesthetic technician; Na = nurse 1; Nb = nurse 2; Sa = consultant surgeon; Sb = junior surgeon) on the position in the sequence of tasks in the procedure. The horizontal line denotes the rank of one (i.e., first in the sequence); a rank of zero means that a participant omitted the task from the card sort as ‘not required’ in the given scenario

Frequency histograms in Fig. 2 show the spread of ranks in the task sequence assigned by individual team members (20 within each of the six member categories) to individual tasks on which there was lowest (57 %–Fig. 2a) and highest average agreement (97 %–Fig. 2b) within OR teams. We also provided an example of a plot showing ranks assigned to a task with a mid-range agreement (80 %–Fig. 2c) for illustrative purposes. The plots demonstrate that the lower the OR team mean similarity score for the sequencing of a task in the procedure, the greater the spread of ranks assigned by team members to that task, with the lowest mean score (in the case of informing the intensive care unit in scenario 1) generating the greatest spread of ranks. By contrast, the highest OR team mean score (in the case of making a surgical incision in scenario 2) had the most unified rankings, regardless of team member. In the case of informing the intensive care unit (Fig. 2a), the majority of surgeons thought this task should be performed at the start of the procedure, while the majority of nurses and anaesthesia subteams believed it should be done sometime in the second half of the procedure.

Table 4 shows the mean percentage of times each subteam believed which of the three possible OR subteams was primarily responsible for a task. On average, the anaesthesia subteam believed they were responsible for ten out of 20 tasks in scenario 1, and nine out of 20 tasks in scenario 2. The surgical subteam believed their subteam was responsible for nine out of 20 tasks in both scenarios, while the nursing subteam believed they were responsible for half the tasks in each scenario. For 14 out of 20 tasks, the majority of each subteam agreed on the same subteam as being primarily responsible for a task. For three tasks (insert urinary catheter; initiate time out; and check drains are turned on), the majority of the subteams agreed that the primary responsibility should be split between the nursing and the surgical subteam. For two tasks (ensure patient warming devices are in place; and provide handover on intraoperative events to PACU staff) responsibility was split between the anaesthesia and the nursing subteam. Subteams produced variable responses for the tasks with lower mean similarity score on responsibilities. For example, all three subteams chose their own subteam as being primarily responsible for checking for optimal patient positioning on the OR table. While the majority of anaesthesia and surgical subteam members agreed that providing handover on intraoperative events to PACU (post-anaesthesia care unit) staff was primarily the responsibility of the anaesthesia subteam, most nurses thought they should be primarily responsible for this task. For confirming estimated blood loss, one of the tasks with lowest similarity scores for both task sequence and responsibility for task, there was also a split within the subteams, with the nurses splitting primary responsibility between all three subteams.

**Table 4:** Mean similarity scores within individual subteams on which subteam (A = anaesthesia subteam; N = nursing subteam; or S = surgical subteam) they rated as primarily responsible for each task. For example, on average, 83 % of the anaesthesia subteam thought their own subteam was primarily responsible for checking blood availability in the two scenarios, while 19 % of the same subteam thought the nurses were primarily responsible, and 1 % thought the surgeons were responsible. Bold figures represent the percentage agreement for the subteam (s) that the majority of participants from all three subteams agreed was primarily responsible for the corresponding task

Task	Who is primarily responsible?^a^								
	Rated by A subteam			Rated by S subteam			Rated by N subteam		
	A	N	S	A	N	S	A	N	S
Check blood availability	**83** %	19 %	1 %	**56** %	21 %	23 %	**63** %	37 %	0 %
Administer anaesthesia induction drugs	**100** %	0 %	0 %	**99** %	0 %	0 %	**99** %	1 %	0 %
Perform a rapid sequence induction	**100** %	0 %	0 %	**99** %	0 %	0 %	**100** %	0 %	0 %
Ensure appropriate antibiotic prophylaxis	**95** %	0 %	5 %	**52** %	1 %	47 %	**84** %	6 %	10 %
Monitor ongoing blood loss	**94** %	4 %	3 %	**63** %	6 %	30 %	**71** %	14 %	15 %
Make surgical incision	0 %	0 %	**100** %	0 %	0 %	**100** %	0 %	0 %	**100** %
Close incision	0 %	1 %	**99** %	0 %	0 %	**100** %	0 %	0 %	**97** %
Remove knife from abdomen (scenario1)	0 %	0 %	**100** %	0 %	3 %	**98** %	0 %	0 %	**100** %
Locate site of perforation (scenario 2)	0 %	3 %	**98** %	0 %	0 %	**100** %	0 %	0 %	**100** %
Fashion stoma (scenario 2)	0 %	0 %	**100** %	0 %	0 %	**100** %	3 %	0 %	**95** %
Initiate sign in	10 %	**85** %	3 %	6 %	**92** %	1 %	15 %	**82** %	3 %
Ensure TED stockings and calf compressors on	1 %	**94** %	4 %	1 %	**87** %	11 %	0 %	**96** %	3 %
Organise bed space in PACU	45 %	**54** %	1 %	25 %	**73** %	1 %	37 %	**61** %	1 %
Initiate sign out	1 %	**83** %	14 %	0 %	**92** %	6 %	0 %	**89** %	10 %
Insert urinary catheter	0 %	**44** %	**56** %	0 %	**14** %	**86** %	0 %	**73** %	**25** %
Initiate time out	1 %	**53** %	**46** %	0 %	**43** %	**57** %	1 %	**61** %	**38** %
Check drains are turned on	0 %	**65** %	**34** %	0 %	**20** %	**77** %	0 %	**73** %	**27** %
Ensure patient warming devices in place	**90** %	**10** %	0 %	**48** %	**48** %	4 %	**44** %	**56** %	0 %
Provide handover on intraoperative events to PACU staff	**71** %	**29** %	0 %	**72** %	**18** %	10 %	**33** %	**66** %	0 %
Check for optimal patient positioning on table	53 %	11 %	36 %	3 %	8 %	90 %	3 %	58 %	39 %
Confirm estimated blood loss	63 %	8 %	30 %	39 %	16 %	44 %	48 %	29 %	23 %
Inform intensive care unit (scenario1)	**73** %	5 %	20 %	40 %	0 %	**60** %	**77** %	13 %	8 %
Remove knife from abdomen (scenario1)	0 %	0 %	**100** %	0 %	3 %	**98** %	0 %	0 %	**100** %
Locate site of perforation (scenario 2)	0 %	3 %	**98** %	0 %	0 %	**100** %	0 %	0 %	**100** %
Fashion stoma (scenario 2)	0 %	0 %	**100** %	0 %	0 %	**100** %	3 %	0 %	**95** %
	**40 %**	26 %	34 %	27 %	25 %	**48 %**	31 %	**37 %**	32 %

^a^For those tasks that some participants believed not to be required in the procedure, the total similarity score for the three OR subteams as rated by a subteam may be less than 100 %

## Discussion

We found that there was poor agreement between OR team members on responsibility for task for half the tasks in each procedure. This has potentially important and concerning implications for safe and efficient team work. OR team members had largely similar understandings of when tasks should be done in an upcoming procedure for all but two tasks, which was more reassuring. Members of the three OR subteams believed their own subteam was primarily responsible for around half the tasks in each procedure.

The relationship between the extent of similarity of mental models within a team and the team’s performance has not been well defined [36]. Furthermore, it is still not clear what constitutes an optimal degree of shared understanding either conceptually [37], or in relation to our similarity scores. Important decisions in an organisational setting presumably warrant a very strong degree of agreement among the decision-makers [38], but there is no empirical evidence to guide the quantification of this in multidisciplinary healthcare teams. It has been suggested that agreement over who is responsible for what may be more important for team performance than agreement over the sequence in which tasks should be done [29]. Redundancy in perceived responsibility for a task may be seen as making that task less likely to be forgotten, but may also result in the (possibly unjustified) assumption that it can be left to others. Furthermore, it would seem to be less efficient to have more than one person taking responsibility for a task, especially in a crisis where time and resources are precious.

Our new Momento tool could be used to identify the potential differences between members of OR teams in their understanding of the key tasks and related responsibilities regarding the upcoming surgical procedure. Making team members aware of the extent of the discrepancies in individual mental models prior to embarking on a case gives them an opportunity to regroup and address the gaps in shared understanding, to make sure all team members are “on the same page” as to who should be responsible for which crucial tasks and when. Providing time for team members to agree, perhaps through a briefing session or in relation to the WHO Surgical Safety Checklist ahead of a procedure, on the order of crucial tasks and on who should be responsible for each task, may help clarify potential ambiguities and better align mental models [39, 40]. The level of disagreement seen in this study for some tasks reinforces an increasing body of evidence supporting a pre-procedure briefing to align understandings and circumvent intra- and postoperative complications and reduce wasted time [41–44].

A secondary outcome of our study is that we have produced a measurement tool for evaluating the similarity of mental models of clinical teams in the OR. This could be easily modified for use in different clinical scenarios. A standard method for evaluating the similarity of mental models that would be applicable in wider research settings has not been reported in literature to date. The choice of a technique to elicit and represent a mental model is considered a key issue in mental model research [45], and the slow progress of empirical work on the sharing of mental models has largely been blamed on the lack of measurement tools [35, 46]. A multi-database search of academic literature, online search engines, and a manual search of associated bibliographies, produced only three studies [31, 34, 35] published in peer-reviewed journals that have combined the ease of use of sorting tasks into a predefined chronological sequence and the ability to simultaneously capture declarative or descriptive knowledge (i.e., the knowledge of what constitutes a task) and procedural knowledge (i.e., knowledge of how to perform the task) [47]. In all three studies, ad hoc teams completed the sorting exercise specifically designed for that study.

A strength of this study lies in the inclusion of different OR professional groups within complete OR teams who have worked together in the past. Mental models have not previously been assessed directly in the context of established, multidisciplinary healthcare teams. The study by Burtscher et al. [31] remains so far the only previous study to quantify mental models in the OR context. These authors, however, only focused on mental models of two-person anaesthesia teams. Larger teams, and especially multidisciplinary ones, may have different dynamics than smaller ones. They engage in more complex processes than smaller teams and they have a greater diversity of view points and expertise due to multiple team members [48–50] Thus, the focus on larger multidisciplinary teams in the current project adds to the previous studies that have focused on mental models of small teams [34, 35], or of only part of the team [31].

Another strength lies in the fact that all teams scored the identical scenario–this was made possible by the use of simulated cases that participants were about to undertake, rather than clinical cases. The fact that participants were preparing to actually manage the cases (albeit in simulation) is likely to have increased their engagement in the process of reading the briefs and responding to the card sorting task, in comparison with the alternative possibility of just providing teams with hypothetical scenarios.

Our card sorting tool, Momento, with modification for particular procedures, could now be used to identify dissimilarities of mental models for various procedures in clinical practice. Given its ease-of-use and customisability, our tool also has the potential to be used in domains reliant on teamwork other than healthcare.

One limitation of our study is that the lack of previous validation of our card sorting tool. While validity was built in to the tool through a rigorous approach to the selection of items for inclusion, further evidence of validity would be desirable.

Other potential limitations of this study include the possibility that there were inaccuracies in card sorting by participants. Also, participants did not have the option of choosing more than one OR subteam as responsible for a task, as the exercise used a forced choice design. The split within OR teams on who should be primarily responsible for certain tasks found in this study might therefore reflect that there should be joint responsibility for those tasks. Future versions of the card sorting exercise should be upgraded to allow for the selection of multiple subteams for those tasks for which there is likely to be joint responsibility.

Our study was limited to cases requiring laparotomy. Future research should extend this work to other types of surgery and surgical specialties.

We measured the similarity of mental models at the beginning of the two cases. Mental models of surgical procedures are likely to be dynamic and the degree of similarity of mental models may well change as cases progress and team members communicate with each other.

In this study we have not established the accuracy of the participants’ mental models. This can be construed as the degree to which these converge with those of guidelines or subject experts, or more generally the degree to which they are grounded in reality. For many tasks it may be more important that team members agree on what should be done, by whom and when, than that this agreement necessarily reflects received wisdom. In some instances however, accuracy in this latter sense probably does matter – and our results may support the idea that agreement tends to be higher in such circumstances. For example, our mean similarity scores were high for who should make the surgical incision and who should administer the anaesthetic drugs.

The extent to which similarity of mental models influences subsequent team performance and patient outcome was beyond the scope of this study and is an area for further research.

## Conclusions

We found differences in the mental models of OR team members about who was responsible for certain tasks, and some variation regarding the order of tasks in an emergency laparotomy. This has implications for effective team function and patient safety. Momento is a tool that could help elucidate and better align the mental models of OR team members about surgical procedures and thereby improve teamwork and outcomes for patients.

## Additional files

Additional file 1: Calculating similarity of mental models. An example considering one team and four tasks is provided to demonstrate in more detail how we calculated the similarity scores for mental models of responsibility for task and similarity of mental models of task sequence. (DOCX 20 kb) Additional file 2: Raw data. Ranks assigned by individual team members (A = anaesthetist; AT = anaesthetic technician; Na = nurse 1; Nb = nurse 2; Sa = consultant surgeon; Sb = junior surgeon) to each of the 20 tasks and a subteam category (A = anaesthesia subteam; N = nursing subteam; or S = surgical subteam) they assigned for responsibility for each task. (DOCX 239 kb)
