# Supplementary material for: Assessing the similarity of mental models of operating room team members and implications for patient safety: a prospective, replicated study
Source: BMC Med Educ. 2016 Aug 31;16(1):229. doi: 10.1186/s12909-016-0752-8 (PMC5007868; doi:10.1186/s12909-016-0752-8)
Supplement: Additional file 1: — Calculating similarity of mental models. An example considering one team and four tasks is provided to demonstrate in more detail how we calculated the similarity scores for mental models of responsibility for task and similarity of mental models of task sequence. (DOCX 20 kb) [file 12909_2016_752_MOESM1_ESM.docx]

# assessing the similarity of mental models of Operating Room Team Members and implications for patient safety: a prospective, replicated study

## Additional file 1 - Calculating similarity of mental models

## Calculating similarity of mental models of responsibility for task

Below is an example considering one team and 4 tasks, for illustrative purposes. Each participant answered which subteam they thought was responsible for the task as ‘A’ (anaesthesia), ‘N’ (nursing), or ‘S’ (surgical). The columns with a grey background are where scores have been calculated.

For example, the anaesthetist and anaesthetic technician agree on who is responsible for Task 1 as they both answered ‘A’ and so the Score for Task 1 is ‘1’. However, there was disagreement between the senior and junior surgeons on who was responsible for Task 3 (one assigned ‘S’ and the other ‘A’), so the surgical subteam’s score for this task is ‘0’. The meam of each of these scores for all twenty surgical (anaesthesia, and nursing) subteams forms the total surgical (anaesthesia, and nursing) subteam score for responsibility for each task. Thus, if there are three OR teams, for example, and anaesthesia subteams from OR team 1, 2 and 3 have a within-team agreement score of 1, 0, and 1 respectively for task 1, and the three surgical subteams have an agreement score of 1, 1, and 1 for the same task, the total similarity score for responsibility for task 1 for anaesthesia team is 0.67 (or 67%), and for surgical team is 1 (i.e., 100%).

| Team 1 |  |  |  |  |  |  |  |  |  |  |  |  |
| --- | --- | --- | --- | --- | --- | --- | --- | --- | --- | --- | --- | --- |
| Subteam | Anaesthesia | | | | Nursing | | | Surgical | | |  |  |
| Participant | Anaesthetist | | Anaesthetic Technician | Score | Nurse 1 | Nurse 2 | Score | Senior | Junior | Score |  | OR team score |
| Task 1 | A | | A | 1 | A | A | 1 | A | A | 1 |  | 100% |
| Task 2 | A | | A | 1 | S | N | 0 | S | S | 1 |  | 26.7% |
| Task 3 | N | | N | 1 | N | N | 1 | S | A | 0 |  | 40% |
| Task 4 | S | | S | 1 | A | A | 1 | S | N | 0 |  | 26.7% |

To calculate the mean similarity score for the OR team, instead of only comparing the answers of participants within a subteam, the mean of all possible pairwise combinations is calculated. Thus, for any given task, the mean of 15 scores is taken, namely

[(A vs AT)+(N1 vs N2)+(S1+S2)+(A vs N1)+(A vs N2)+(A vs S1)+(A vs S2)+(AT vs N1)+(AT vs N2)+(AT vs S1)+(AT vs S2)+(N1 vs S1)+(N1 vs S2)+(N2 vs S1)+(N2 vs S2)]

In the example above, we can compute the OR team score for task 2 as

[(1)+(0)+(1)+(0)+(0)+(0)+(0)+(0)+(0)+(0)+(0)+(1)+(1)+(0)+(0)]/15 = 0.267 (or 26.7% agreement)

As with subteams, the total OR team similarity score for responsibility for task is the mean of the twenty OR team scores for each task.

## Calculating similarity of mental models of task sequence

In the case of task sequence, the participants ranked the tasks so the numeric value that is their score is the position in the list of twenty tasks. Participants had an option to assign the same rank to two or more tasks if they believed those tasks should be performed at the same time during the procedure. To account for tied ranks, we reranked individual ranks so that the tied observations each received the lowest possible rank over all such observations. For example, if task A ranked ahead of tasks B and C (which compare equal) which were both ranked ahead of D, then A got ranking number 1 ("first"), B got ranking number 2 ("joint second"), C also got ranking number 2 ("joint second") and D got ranking number 4 ("fourth"). Tasks considered “not required” by participants were assigned a “NULL” rank.

To calculate the similarity scores for task sequence, rather than assignment of responsibilities, instead of a binary score for agreement (‘1’ or ‘0’) we used the distance between the task position or rank in the list. To illustrate this, the same team and four example tasks are shown below. For example, the senior surgeon thought that task 1 would occur third, whereas the junior surgeon thought it would come first. Therefore, there is a difference of 2. If there was a task that was ranked as “NULL”, because it was considered “not required”, then the distance was set to the maximum possible distance (in this case, 3). However, the difference is a measure of how far away the scores are. In order to convert these to a measure of similarity, we first work out the maximum possible distance score. In this example, because there are 4 tasks, the maximum possible difference is 3 for any given task. The relative distance is then distance score divided by the maximum possible distance (3 in this case). To scale between a score of 1 to represent total agreement and a score of 0 for total disagreement, we must subtract the relative distance from 1. For example, the distance between the senior and junior surgeons for task 1 is 2. Thus, the surgical team score for task 1 is [1-(2/3) = 0.333 or 33.3% agreement or similarity]. The mean of each of the twenty surgical subteams’ scores for a task forms the total similarity score for task sequence for the surgical subteam for that task. Similarly, the mean of all twenty anaesthesia/nursing subteams’ scores for task 1 represents the total anaesthesia/nursing subteam similarity score for task sequence for task 1, etc.

| Team 1 |  |  |  |  |  |  |  |  |  |  |  |  |  |  |
| --- | --- | --- | --- | --- | --- | --- | --- | --- | --- | --- | --- | --- | --- | --- |
| Subteam | Anaesthesia | | |  | Nursing | | |  | Surgical | | |  |  |  |
| Participant | Anaesthetist | Anaesthetic Technician | Distance | Score | Nurse 1 | Nurse 2 | Distance | Score | Senior | Junior | Distance | Score |  | OR team score |
| Task 1 | 1 | 4 | 3 | 0 | 1 | 2 | 1 | 0.667 | 3 | 1 | 2 | 0.333 |  | 51.1% |
| Task 2 | 2 | 1 | 1 | 0.667 | 3 | 1 | 2 | 0.333 | 2 | 3 | 1 | 0.667 |  | 64.4% |
| Task 3 | 3 | 3 | 0 | 1 | 2 | 4 | 2 | 0.333 | 1 | 4 | 3 | 0 |  | 53.3% |
| Task 4 | 4 | 2 | 2 | 0.333 | 4 | 3 | 1 | 0.667 | 4 | 2 | 2 | 0.333 |  | 62.2% |

In a similar way to the similarity on responsibility for task, we calculate the mean OR team similarity score for a task by first computing the mean similarity score for every combination of participants within that OR team. In this case, for task 2, we have:

[(0.67)+(0.33)+(0.67)+(0.67)+(0.67)+(1)+(0.67)+(0.33)+(1)+(0.67)+(0.33)+(0.67)+(1)+(0.67)+(0.33)]/15 = 0.644, or 64.4% agreement.

The total OR team similarity score for task sequence for a task is then the mean of twenty OR teams’ scores for that task.
