# Supplementary material for: Assessing the similarity of mental models of operating room team members and implications for patient safety: a prospective, replicated study
Source: BMC Med Educ. 2016 Aug 31;16(1):229. doi: 10.1186/s12909-016-0752-8 (PMC5007868; doi:10.1186/s12909-016-0752-8)
Supplement: Additional file 2: — Raw data. Ranks assigned by individual team members (A = anaesthetist; AT = anaesthetic technician; Na = nurse 1; Nb = nurse 2; Sa = consultant surgeon; Sb = junior surgeon) to each of the 20 tasks and a subteam category (A = anaesthesia subteam; N = nursing subteam; or S = surgical subteam) they assigned for responsibility for each task. (DOCX 239 kb) [file 12909_2016_752_MOESM2_ESM.docx]

# assessing the similarity of mental models of Operating Room Team Members and implications for patient safety: a prospective, replicated study

*Additional file 2 – Raw data*
